# Evaluation of Current Symptoms in Postoperative Achilles Tendons: A Multimodal Ultrasound Study

**DOI:** 10.3390/healthcare9030288

**Published:** 2021-03-05

**Authors:** Priscila Nunes, Marcel Betsch, Bernhard Fuss, Timm Dirrichs, Markus Tingart, Valentin Quack, Matthias Gatz

**Affiliations:** 1 Department of Orthopedics, University Hospital RWTH Aachen, 52074 Aachen, Germany; mtingart@ukaachen.de (M.T.); vquack@ukaachen.de (V.Q.); mgatz@ukaachen.de (M.G.); 2 Department of General and Visceral Surgery, St. Elisabeth Hospital, 52511 Geilenkirchen, Germany; 3 Department of Orthopedic and Trauma Surgery, University Hospital Mannheim, 68167 Mannheim, Germany; marcel.betsch@gmx.de; 4 Praxisklinik Orthopädie, University Hospital Aachen Franziskus, 52074 Aachen, Germany; fuss@pko.ac; 5 Department of Diagnostic and Interventional Radiology, University Hospital RWTH Aachen, 52074 Aachen, Germany; tdirrichs@ukaachen.de; 6 Department of Trauma and Reconstructive Surgery, University Hospital RWTH Aachen,52074 Aachen, Germany

**Keywords:** postoperative, Shear Wave Elastography, Ultrasound Tissue Characterization, Power Doppler, Achilles tendon, surgery

## Abstract

(1) Background: It is unknown which imaging parameters are associated with clinical persistent symptoms in postoperative Achilles tendons. This study used B-Mode, Power Doppler (PD-US), Ultrasound Tissue Characterization (UTC) and Shear Wave Elastography (SWE) to investigate which imaging parameters are associated with persistent symptoms in postoperative Achilles tendon tissue. (2) Methods: Retrospective, cross-sectional, multimodal imaging study. Based on the VISA-A score, postoperative tendons were assigned to two groups: 1. asymptomatic (VISA-A ≥ 90, *n* = 18); 2. symptomatic (VISA-A < 90, *n* = 10). The following imaging parameters were analyzed: UTC (echo type I, II, III, IV), B-Mode (diameter, cross sectional area, calcification, fiber irregularity), PD-US (Öhberg score) and SWE (SWE 3 mm, SWE area) using a *t*-test and a *Mann–Whitney U* test. (3) Results: SWE and PD-US showed significantly reduced elasticity and increased neovascularization in symptomatic tendons (SWE 3 mm *p* = 0.031, SWE area *p* = 0.046, Öhberg score *p* < 0.001). The only significant correlation between imaging parameters and the VISA-A score was assessed for SWE 3 mm (r = 0.378; *p* = 0.047) and the Öhberg score (r = −0.737; *p* < 0.001). Conclusions: Symptomatic postoperative Achilles tendons showed increased neovascularization and lower SWE values than asymptomatic ones. Future studies should examine the diagnostic accuracy of PD-US and SWE in detecting current symptoms in postoperative Achilles tendons.

## 1. Introduction

Achilles tendon ruptures and open surgical procedures such as tendon detachment/reattachment procedures for Achilles tendinopathies may cause high tissue damage within the Achilles tendon. Even after surgical management of Achilles tendon tears and tendinopathies, persistent pathological ultrasound findings such as areas of hypoechogenicity, fiber irregularity and tendon thickening can be found in asymptomatic patients [1,2,3]. Currently, it is unclear which imaging findings correlate with persistent symptoms in postoperatively altered tendon tissue [4]. The gold standard to detect Achilles tendon pathologies is still the B-Mode and Power Doppler (PD-US) ultrasound, but there is a low correlation of symptoms with pathological ultrasound findings [4,5,6]. Therefore, it is questionable whether the conventional ultrasound modalities such as B-Mode and PD-US can adequately detect clinical symptoms after traumatic lesions caused either by trauma or the surgical approach [4,7]. In recent years, technological advancements such as Ultrasound Tissue Characterization (UTC) and Shear Wave Elastography (SWE) have been introduced [8,9]. In particular, SWE, which provides information about tissue elasticity, shows promising results for the evaluation of structural alterations in the follow up of Achilles tendinopathy and after surgery [7,10,11,12]. Zhang et al. showed in their study that repaired tendons after rupture gradually increase in their elasticity postoperatively, and that these findings correlate with improved AOFAS scores [10]. Moreover, Frankewycz et al. reported that the elastic Achilles tendon properties still remain decreased after 2 years compared with the contralateral non-injured side [11]. In the current literature, it has not been examined whether SWE is able to differentiate between symptomatic and asymptomatic postoperative Achilles tendons. Furthermore, for UTC, which provides semi-histological data based on an algorithm analyzing ultrasound signals, there are no studies currently available that have used this technology to evaluate postoperative tendons [8]. In general, most current studies focus on the evaluation of the tendon structure in correlation to the postoperative timing, without considering current clinical symptoms. The purpose of this diagnostic imaging study was to investigate whether UTC and SWE have further value in the evaluation of postsurgical and post-traumatic Achilles tendons. We hypothesized that UTC and SWE are able to distinguish between symptomatic and asymptomatic postsurgical Achilles tendons.

## 2. Materials and Methods

This is a retrospective, cross-sectional, multimodal diagnostic study, which evaluates the association of imaging parameters with clinical symptoms in postoperative Achilles tendons using modern ultrasound technologies. This study was approved by the ethics committee of the local medical university and all participants provided written informed consent (EK 232/17).

### 2.1. Patients

**Figure 1:**
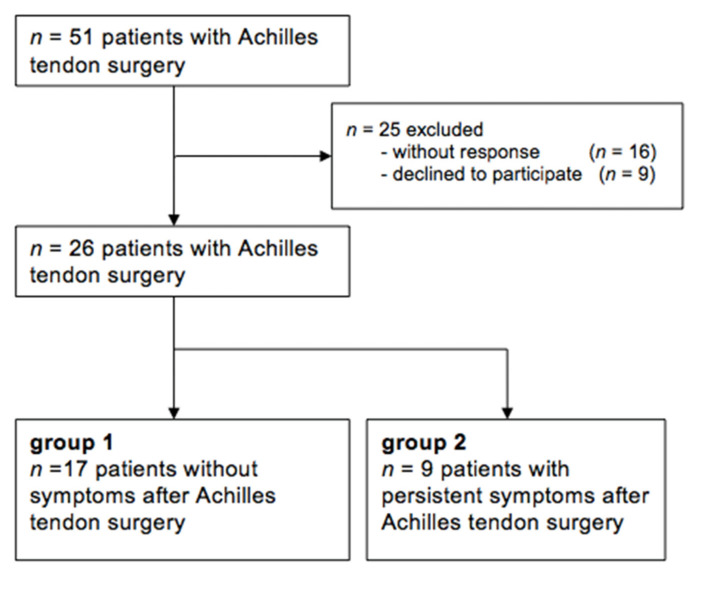
Flow chart of the study design.

The institutional database was scanned in December 2017 for cases of open Achilles tendon surgeries, revealing *n* = 51 procedures in the previous five years. Patients were contacted by telephone and letters to participate in this study, and ultrasound examinations were performed between June 2018 and March 2019 (Figure 1). Inclusion criteria were the following: surgical procedures after Achilles tendon rupture or recalcitrant Achilles tendinopathy such as (a) tendon detachment and reattachment for insertional Achilles tendinopathy, (b) tendon reinsertion with anchors for insertional rupture, (c) open repair for midportion Achilles tendon ruptures, and (d) longitudinal splitting and excision of tendinopathic areas in midportion Achilles tendinopathy. Further inclusion criteria were age ≥18 years, a minimum postoperative time of three months, no pregnancy, not over- or underweight (BMI > 35; BMI < 17), no additional surgical procedure at the foot and ankle region, and no peritendinous injections in the previous six months. Refusal of study participation and no response were defined as exclusion criteria.

All evaluated Achilles tendons were assigned to one of the following two cohorts based on the current clinical symptoms and evaluated with the Victorian Institute of Sports Assessment (VISA-A) score (range 0–100; asymptomatic 100 points) [13,14] independently from the underlying surgical procedure (Figure 1). Based on the study of Iversen et al. a VISA-A score ≥ 90 was chosen to be the cut-off value, representing an excellent and asymptomatic outcome [13]:

- Group 1: VISA-A score > = 90 representing asymptomatic Achilles tendons;
- Group 2: VISA-A score < 90 representing symptomatic Achilles tendons.

Furthermore, the American Orthopedic Foot and Ankle Society Score (AOFAS) (range 0–100, asymptomatic 100 points) [15] and the Roles and Maudesly score (excellent, good, acceptable, poor) were used to rate the clinical symptoms. General symptom improvement compared to the presurgical status was assessed with a 6-point Likert scale (completely recovered, much improved, somewhat improved, hardly improved, not improved, worse).

### 2.2. Data Acquisition with B-Mode, PD-US and SWE

Participants underwent a standardized multimodal ultrasound protocol based on B-Mode, PD-US and SWE of the operated Achilles tendon. For the examinations, a high-resolution linear 18 MHz transducer was used (AixplorerTM, SuperLinearTM SL 18-4, Supersonic Imagine, Aix-en-Provence, France). During the examination of the Achilles tendon, patients were lying in a prone position with their foot hanging over the examination table. A gel-cushion delay block (Sonogel, Sonokit Proxon, area 100 mm × 100 mm, delay distance 10 mm) was used to improve docking.

**Figure 2:**
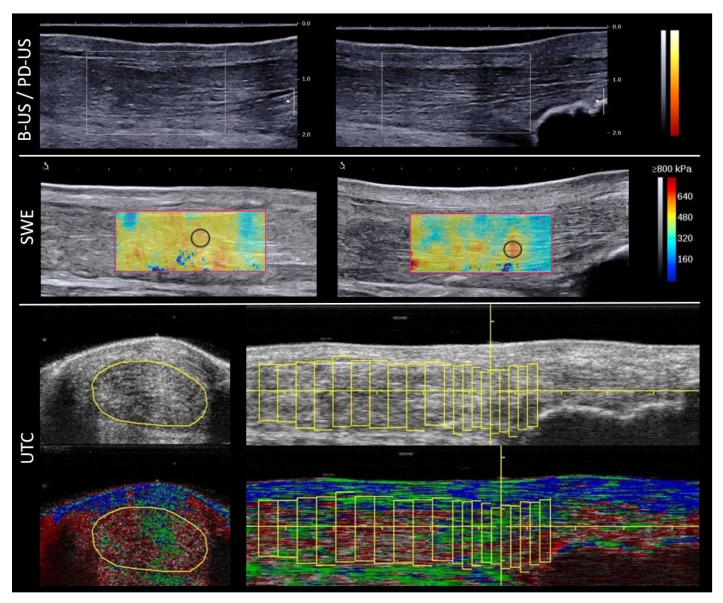
B-Mode (B-US), Power Doppler (PD-US), Shear Wave Elastography (SWE) and Ultrasound Tissue Characterization (UTC): asymptomatic patient 12 months after midportion rupture and suture. There is no neovascularization, but focal hypoechogenicity and a thickened tendon in B-US and PD-US. For the insertion, SWE revealed high elastic properties (SWE 3 mm (black circle): 538.4 kPa (13.38 m/s) SD 12.3 kPa (2.02. m/s); SWE area (red rectangle): 469.5 kPa (12.49 m/s) SD 78.3 kPa (5.1 m/s), area 2.44 cm2). UTC shows a large area of echo type III (red—fibrillar matrix) in the midportion in the axial and longitudinal planes. UTC echo type I—intact and aligned tendon bundles (green); UTC echo type II—discontinuous wavy tendon bundles (blue); UTC echo type III—mainly fibrillar matrix (red); UTC echo type IV—mainly amorphous matrix (black).

The anterior–posterior diameter of the Achilles tendon was measured in the longitudinal and transverse planes. Cross-sectional area (CSA) was evaluated in the transverse plane at the thickest point of the tendon in order to achieve a strictly orthogonal scan through the oval tendon. Furthermore, the tendon was assessed for the appearance of hypoechogenicity, fascicle irregularity, peritendinous fluid, calcification and bursitis. PD-US evaluated the amount of detectable neovascularization and the findings were rated with the Öhberg score (0 = no vessels; 1 = one or two vessels anterior of the tendon; 2–4: two, three, four or more vessels inside the tendon) [16] (Figure 2).

SWE delivers quantitative parameters of the tissues’ mechanical properties by measuring shear wave speed propagation (m/s) and by deducing the Young’s modulus (kPa) with a high reliability (inter-observer: 0.940, intra-observer: 0.916) [8,17]. The obtained SWE information was rated quantitatively in Kilopascals (kPa), up to a maximum tissue rigidity of 800 kPa (Figure 2). To reduce spatial-dependent data influence, the Region of Interest (ROI) for measurements was a circle of 3 mm in diameter with data acquisition in the most rigid part of the tendon (SWE 3 mm) and an individual area (up to 0.625 cm^2^) covering the whole tendon in the SWE measurement window (SWE area) (Figure 2). For both the insertion and midportion, SWE measurements (*n* = 6) were conducted three times and a mean value was calculated [7,9].

### 2.3. UTC Data Acquisation

UTC evaluates the consistency of grey level in successive transverse tendon images and generates images in the sagittal, coronal and transverse planes. The tendon images are captured by standardized transducer tilt angle, focus depth and ultrasound gain. Therefore, UTC is thought to be more user independent and provides an excellent inter-observer reliability for Achilles tendons (0.92–0.95) [18]. UTC data acquisition was based on previously published standardized protocols [18,19,20]: patients were in a prone position with their foot hanging over the examination couch with a maximal dorsiflexion of the ankle [21]. For the acquisition of images, a 7–10 MHz linear ultrasound transducer was used (SmartProbe 12L5-V, Terason 2000+; Teratech; Burlington, MD, USA), which was positioned in a tracking device (UTC Tracker, UTC Imaging, Stein, The Netherlands). The tracker with the applied ultrasound probe moves automatically along the tendon longitudinal axis over a distance of 12 cm. Hereby, regular axial images at intervals of 0.2 mm from proximal to distal were recorded.

Tendon structure was quantified with a Region of Interest covering the CSA of the tendon in the axial plane. Starting from the proximal border of the calcaneus, the insertion of the Achilles tendon was contoured with ROIs at intervals of 2 mm (every tenth image) covering 2 cm and the midportion at intervals of 4 mm (every twentieth image) covering 2 cm above the calcaneus to 6 cm proximal. Using the standardized UTC algorithm (17 continuous images), tendons were classified into four echo types giving semi-histological information [18] (Figure 2):

- Echo type I—intact and aligned tendon bundles (green);
- Echo type II—discontinuous wavy tendon bundles (blue);
- Echo type III—mainly fibrillar matrix (red);
- Echo type IV—mainly amorphous matrix (black).

### 2.4. Statistical Analysis

For all analyses, SPSS 24.0 (IBM, Chicago, US) was used to assess statistical significance, which was defined as *p* < 0.05. Descriptive statistics with means and standard deviations (SD) were used. Normal distribution was evaluated with a Kolmogorov–Smirnov test. Significant differences between the two groups were assessed with a *t*-test and a *Mann–Whitney U* test. Correlations between ultrasound parameters and score values were assessed with the Pearson correlation coefficient. ANCOVA was used to examine the influence of the current symptoms and factors such as BMI, age, postoperative time and operation technique on imaging parameters.

## 3. Results

Figure 1 demonstrates the study design, with in total *n* = 18 asymptomatic and *n* = 10 symptomatic included tendons. Patients´ demographics and the distribution of surgical procedures are shown in Table 1. There was no significant difference concerning BMI, age and postoperative time between the two groups (Table 1). An ANCOVA demonstrated that the imaging parameters were significantly influenced by the VISA-A score value, but there was no influence of BMI, age, postoperative time, underlying etiology and operation technique on imaging parameters.

**Figure 3:**
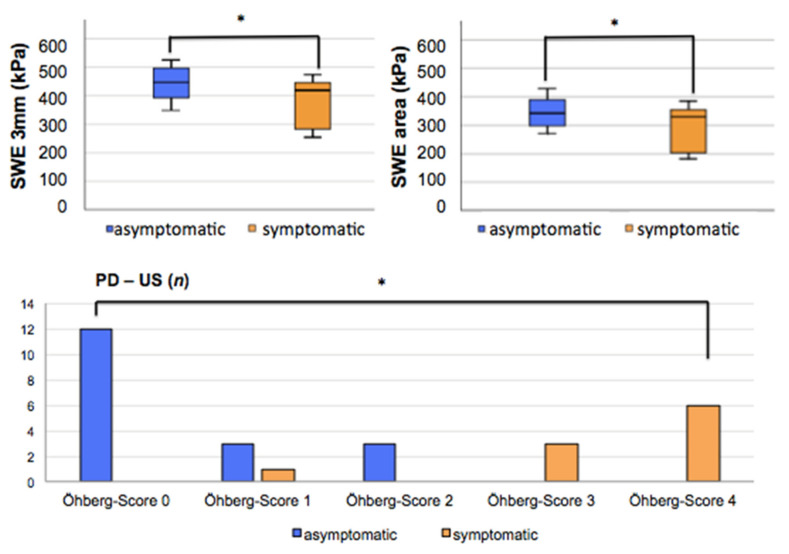
Significant differences (*) between asymptomatic and symptomatic postoperative tendons could only be demonstrated in SWE 3 mm, SWE area and PD-US.

Table 2 summarizes the ultrasound parameters of B-Mode, PD-US, SWE and UTC measurements. There was no significant difference concerning B-Mode parameters between asymptomatic and symptomatic postoperative tendons (*p* = 0.226–1.00). Moreover, UTC could also not find any significant differences between the groups (*p* = 0.265–0.993). However, SWE was able to depict a significant higher tendon elasticity in asymptomatic tendons compared to symptomatic tendons (*p* = 0.031, *p* = 0.046). Additionally, PD-US revealed a significantly lower Öhberg score in postoperative tendons with no clinical symptoms (*p* < 0.001) (Figure 3).

Based on these results, the only significant correlation between imaging parameters and the VISA-A score was found for SWE 3 mm (r = 0.378; *p* = 0.047) and for the Öhberg score (r = −0.737; *p* < 0.001). Additionally, the AOFAS score correlated significantly to SWE 3 mm (r = 0.436; *p* = 0.021) and SWE area (r = 0.391; *p* = 0.040).

## 4. Discussion

This pilot study comparatively examined imaging parameters of B-Mode, PD-US, UTC and SWE between symptomatic and asymptomatic postoperative Achilles tendons that were treated with an open surgical approach. The results of our study suggest that B-Mode and UTC cannot differentiate between asymptomatic and symptomatic postoperative tendons, in contrast to SWE or PD-US, which showed higher SWE elastic properties and a lower degree of neovascularization in asymptomatic tendons. Previous SWE studies found higher elastic properties in asymptomatic tendons compared to symptomatic ones and an increase in elastic properties in line with symptom reduction in various tendinopathies and in plantar fasciitis [7,9,22]. However, there might be a persistent reduction in Achilles tendon elastic properties in comparison with the contralateral non-injured side as reported by Frankewycz et al. [11]. Additionally, Zhang et al. showed that repaired tendons after rupture gradually increase in their elasticity postoperatively, with a representative correlation of SWE values to AOFAS score values [10]. In this sense, the present study confirmed a correlation of the VISA-A and AOFAS scores with SWE values, although the correlation coefficient was low. Even though histopathological analyses of the examined tendons are missing in the present study, our findings might be indirectly based on a reduced edema within the asymptomatic tendons, so that SWE could display higher elastic properties [23]. Considering the current literature, tendon biopsies confirmed that tendinopathic tissue is associated with a higher concentration of water binding proteoglycans such as Versican and Aggrecan, which might induce edematous swelling and symptoms [23]. This might be relevant for a better understanding of tendon healing. Considering the continuum model and previous studies, the integrity of the collagen fascicles after trauma or tendinopathy might not be directly associated with the improvement or persistence of symptoms [24,25,26]. This could be also confirmed by our study, which did not show a significant difference in the semi-histological data sets of UTC echo types of asymptomatic and symptomatic postoperative tendons. Unfortunately, further comparative postoperative UTC data are missing in the literature, so that a final conclusion of the value of UTC for postoperative care is limited. However it has to be considered that the UTC algorithm is indirectly based on B-Mode findings.

According to B-Mode parameters, the literature reveals a round tendon configuration in the cross-sectional area, calcification, loss of the fibrillar pattern, unregular borders of the paratenon and persistent tendon thickness of approximately 10 mm after a surgically treated rupture, which does not return to baseline in long-term follow up [1,2,3,27,28]. In general, correlation of grey scale imaging findings to clinical symptoms and function is low, which could be confirmed by the present study revealing no significant differences between symptomatic and asymptomatic postoperative tendons in any of the B-Mode parameters [4,5,6,28]. Therefore, experienced interpretation is required to not misinterpret findings [4,5,6,28]. Furthermore, the lack of restitution ad integrum is commonly observed in asymptomatic tendons, because symptoms might not be only based on pathological imaging findings, but rather on non-structural factors, which might not be adequately assessed with conventional ultrasound technologies such as B-Mode [4]. However, some authors observed that after 3 months, tendon thickness begins to decrease over time, collagen fiber orientation improves and neovascularization and adhesions decrease [1,29]. Interestingly, the Achilles tendons of newborns with congenital clubfoot treated with percutaneous tenotomy have a strong healing potential with only a persistent mild thickening and rearranged collagen fascicles after 6–12 months, which might highlight the high potential of stem cell therapy and a possible restitution ad integrum in immature tissue [30].

Current literature indicates that neovascularization might be increased during the initial healing process up to 6 months, followed by a stabilization and regression of pathological neovascularization in the postoperative course until the avascular scare tissue is formed [27,28,29,31,32]. However, this is primarily based on expert opinions or literature reviews based on insufficient primary data without considering a semi-quantitative score assessment such as the Öhberg score [27,28,29,31,32]. Shih et al. reported in their study (*n* = 10) an absence of neovascularization 6 years after surgical repair [33]. Miquel et al. observed that neovascularization was detectable in 10/17 Achilles tendons treated with percutaneous repair and that the degree of detectable neovascularization (high, moderate, low) decreases depending on the postoperative time, with only low presence of neovascularization after 12–24 months [34]. However, an ANCOVA analysis revealed no significant influence of the postoperative time on the amount of neovascularization in our study cohort. Nevertheless, the presence of neovascularization needs to be interpreted with caution, since neovascularization might also be present in asymptomatic individuals with high loads and there is no correlation of neovascularization with clinical scores in patients suffering from tendinopathies [9,35]. In contrast, the present study detected a highly negative correlation of neovascularization with clinical scores and a significant difference between asymptomatic and symptomatic postoperative tendons. Thus, future studies need to clarify the value of neovascularization scoring in postoperatively treated symptomatic Achilles tendons.

The strength of the present study is the comparative approach evaluating four different ultrasound modalities. However, the results of our study need to be interpreted in light of the following limitations. Several cofactors such as different preoperative diagnoses, surgical techniques or postoperative time points might have slightly biased our findings, even though an ANCOVA analysis revealed that the presence of postoperative symptoms was the only factor with a significant influence on our findings. The missing differentiation between the lesions caused by traumatic rupture with frayed tendon stumps or by sharp surgical dissection of tendinopathic tissue for tendon detachment in particular might have influenced the validity of our data. However, the rupture itself and/or an open surgical procedure of underlying Achilles tendinopathy cause tissue damage and the present study is rather a pilot study with a small sample size aiming to investigate which one of the various available ultrasound modalities has the highest potential to depict current symptoms in postoperatively altered tendons. Additionally, there might be a possible selection bias, as nearly half of the patients refused study participation. Furthermore, current symptoms were assessed with the AOFAS score as a general foot and ankle score and the VISA-A score, which is primarily used for non-operatively treated Achilles tendinopathy. This could have influenced symptom assessment especially after Achilles tendon rupture.

## 5. Conclusions

Symptomatic postoperative Achilles tendons showed significantly higher neovascularization and lower SWE values than asymptomatic ones. B-Mode and UTC might have no further value for symptom detecting in postoperative care. Future studies should examine the diagnostic accuracy of PD-US and SWE and the influence of further cofactors such as the surgical technique or the postoperative time on imaging parameters.

## Figures and Tables

**Table 1:** Demographic data (range and standard deviation (SD)) of the asymptomatic and symptomatic cohort.

Parameter	Asymptomatic: *n* = 17 Participants *n* = 18 Tendons	Symptomatic: *n* = 9 Participants *n* = 10 Tendons	Significance
Insertional AT (tendons)	5	6	
Insertional rupture (tendons)	1	0	
Mid-portion AT (tendons)	4	2	
Mid-portion rupture (tendons)	8	2	
Male (%)	83.3	44.4	
Age (years)	55 (29–79; SD 14.5)	53 (44–61; SD 5.9)	*t*(24) = 0.258, *p* = 0.799
Sport (hours/week)	3.0 (2–4; SD 0.9) *n* = 6	0	
Body mass index (kg/m^2^)	26.3 (23–30; SD 2.3)	29.4 (23–40; SD 7.4)	*t*(24) = −1.629, *p* = 0.116
Postoperative time (months)	25 (6–60; SD 13)	21 (3–60; SD 17)	*t*(24) = 0.607, *p* = 0.550
Months until symptom relief	7.6 (3–14; SD 4)		
VISA-A score	98 (90–100; SD 3)	48 (22–85; SD 25)	*t*(24) = 8.267, *p* < 0.001
AOFAS score	97 (90–100; SD 5)	76 (69–85; SD 5)	*t*(24) = 10.642, *p* < 0.001
Roles and Maudesly score (*n*)—excellent/good/acceptable/poor	7/10/0/0	0/0/3/6	
Likert scale—completely recovered/much improved/somewhat improved/hardly improved/not improved/worse	11/6/0/0/ 0/0	0/2/4/3/ 0/0	

One patient of each group had a bilateral operation for insertional AT. Victorian Institute of Sports Assessment (VISA-A). American Orthopedic Foot and Ankle Society Score (AOFAS).

**Table 2:** Comparison between asymptomatic and symptomatic postoperative tendons.

Modality	Parameter	Asymptomatic Postoperative	Symptomatic Postoperative	Significance
B-US	thickness longitudinal (cm) thickness transverse (cm) cross-sectional area (cm^2^) hypoechogenicity fascicle irregularity peritendinous fluid calcification bursitis	1.1; SD 0.25	1.0; SD 0.38	*t*(26) = 0.215, *p* = 0.831
		1.1; SD 0.21	1.1; SD 0.42	*t*(26) = −0.232, *p* = 0.818
		1.8; SD 0.84 *n* = 16 *n* = 18 *n* = 0 *n* = 2 *n* = 3	2.0; SD 0.9 *n* = 10 *n* = 10 *n* = 2 *n* = 4 *n* = 2	*t*(26) = −0.46, *p* = 0.646 *U* = 80, *p* = 0.654 *U* = 90, *p* = 1.000 *U* = 72, *p* = 0.408 *U* = 64, *p* = 0.226 *U* = 87, *p* = 0.906
PD-US	Öhberg score 0 Öhberg score 1 Öhberg score 2 Öhberg score 3 Öhberg score 4	*n* = 12 *n* = 3 *n* = 3 *n* = 0 *n* = 0	*n* = 0 *n* = 1 *n* = 0 *n* = 3 *n* = 6	*t*(26) = −8.624, *p* < 0.001 *
SWE	SWE 3 mm (kPa) (m/s) SWE area (kPa) (m/s)	443.7 (357–52; SD 55) 12.1; SD 0.8	384.6 (253–478; SD 83) 11.3; SD 1.2	*t*(26) = 2.277, *p* = 0.031 *
		347.4 (281–428; SD 49) 10.8; SD 0.8	298.6 (194–383; SD 75) 9.97; SD 1.2	*t*(26 = 2.098, *p* = 0.046 *
UTC	echo type 1 echo type 2 echo type 3 echo type 4	27.4 (14–47; SD 10)	29.4 (11–59; SD 18)	*t*(24) = −0.383, *p* = 0.705
		19.9 (13–26; SD 4)	19.9 (10-32; SD 8)	*t*(24) = 0.008, *p* = 0.993
		32.3 (17–48; SD 8)	33.4 (9–53; SD 16)	*t*(24) = −0.222, *p* = 0.826
		20.4 (12–27; SD 5)	17.3 (4–28; SD 9)	*t*(24) = 1.142, *p* = 0.265

Significant difference *p* < 0.05 has been marked *. Due to hardware problems, only 16 tendons could be included for the UTC scan in the asymptomatic group. Mean values, range and standard deviation (SD) are given. B-Mode (B-US), Power Doppler (PD-US), Shear Wave Elastography (SWE) and Ultrasound Tissue Characterization (UTC).

## Data Availability

The data presented in this study are available on request from the corresponding author. The data are not publicly available due to data restriction policies.
